# Perforated Meckel's diverticulitis complicating active Crohn's ileitis: a case report

**DOI:** 10.1186/1752-1947-3-12

**Published:** 2009-01-13

**Authors:** Frank Schwenter, Pascal Gervaz, Philippe de Saussure, Thomas McKee, Philippe Morel

**Affiliations:** 1 Department of Surgery, University Hospital and Medical School Geneva, 1211 Geneva 14, Switzerland; 2 Department of Gastroenterology, University Hospital and Medical School Geneva, 1211 Geneva 14, Switzerland; 3 Department of Pathology, University Hospital and Medical School Geneva, 1211 Geneva 14, Switzerland

## Abstract

**Introduction:**

In Crohn's disease, the extension of active terminal ileitis into a Meckel's diverticulum is possible, but usually has no impact on clinical decision-making. We describe an original surgical approach in a young woman presenting with a combination of perforated Meckel's diverticulitis and active Crohn's ileitis.

**Case presentation:**

We report the case of a 22-year-old woman with Crohn's disease, who was admitted for abdominal pain, fever and diarrhoea. CT scan demonstrated active inflammation of the terminal ileum, as well as a fluid collection in the right iliac fossa, suggesting intestinal perforation. Laparoscopy was performed and revealed, in addition to extensive ileitis, a 3 × 3 cm abscess in connection with perforated Meckel's diverticulitis. It was therefore possible to avoid ileocaecal resection by only performing Meckel's diverticulectomy; pathological examination of the surgical specimen revealed the presence of transmural inflammation with granulomas and perforation of the diverticulum at its extremity.

**Conclusion:**

Crohn's disease of the ileum may be responsible for Meckel's diverticulitis and cause perforation which, in this case, proved to be a blessing in disguise and spared the patient an extensive small bowel resection.

## Introduction

The prevalence of Meckel's diverticulum in patients with Crohn's disease is probably similar to the general population, although some authors have reported an increased (5.8%) frequency [1]. Extension of the inflammatory process into the diverticulum is uncommon, and very few inflammatory bowel disease patients will develop complications specifically related to Meckel's diverticulitis [2,3]. There is, however, evidence that ileal Crohn's lesions may spread to Meckel's diverticulum, resulting in diverticulitis [4], associated with small bowel obstruction [5] or enterovesical fistula [6]. We describe herein the surgical management of a young woman with Crohn's ileitis, who developed inflammation and eventually perforation in a Meckel's diverticulum.

## Case presentation

**Figure 1:**
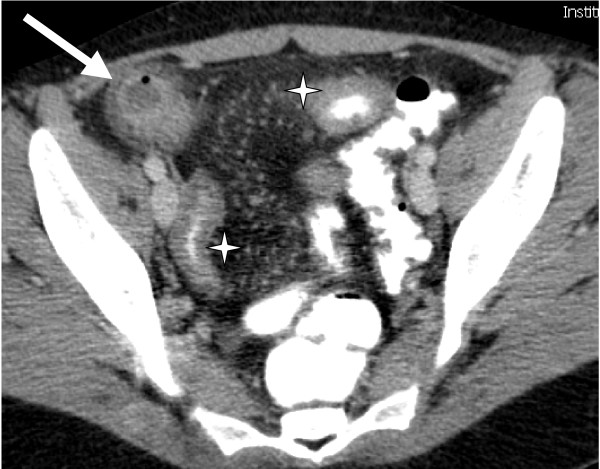
Preoperative Computed Tomography scanner showing a 3 × 3 cm abscess in right iliac fossa (arrow) as well as extensive inflammation of the terminal ileum (*).

A 22-year-old Caucasian woman was admitted because of severe abdominal pain, fever and diarrhoea. She had been diagnosed with Crohn's disease 2 months before, and a computed tomography (CT) scan demonstrated active inflammation of the terminal ileum, as well as a 3 × 3 cm abscess in the right iliac fossa (Figure 1). The initial management was conservative, with metronidazole 500 mg TID, ciprofloxacin 500 mg twice daily (BD), azathioprine 150 mg once daily (OD) and percutaneous CT scan-guided drainage of the abscess. This proved unsuccessful and surgery was considered following the development of persistent purulent drainage from the drain orifice.

**Figure 2:**
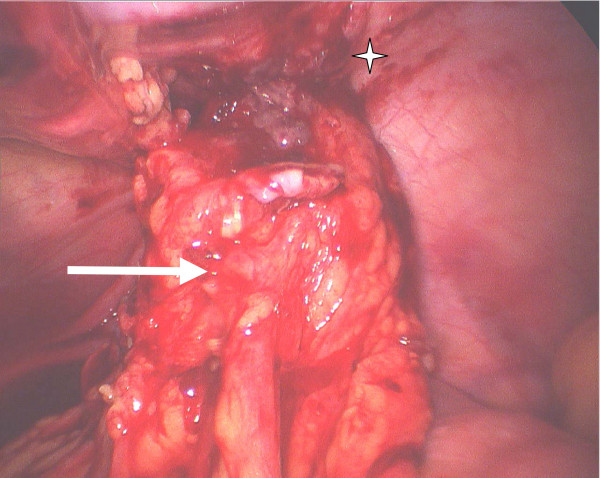
Laparoscopic approach demonstrating an inflammatory mass corresponding to the Meckel's diverticulum (arrow) adherent to the abdominal wall (*).

**Figure 3:**
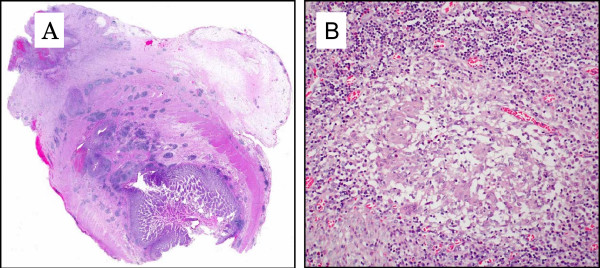
**Histology of resected Meckel's diverticulum: (A) active chronic transmural inflammation with micro-abscesses and granulomas (×40); (B) enlarged view (×400) of a giant cell granuloma**.

Laparoscopy was performed, and revealed features typical of extensive small bowel Crohn's disease, involving the last 80 cm of the ileum, as well as a fistulising 3 × 3 cm abscess adherent to the anterior abdominal wall. The origin of the abscess proved to be a perforated Meckel's diverticulum (Figure 2). A conservative surgical option was preferred in order to avoid an extensive bowel resection, and Meckel's diverticulectomy was performed using an endoGIA stapler fired at the base of the diverticulum. Pathological examination of the surgical specimen revealed the presence of an active transmural inflammation with granulomas and perforation of the diverticulum at its extremity (Figure 3).

The postoperative course was uneventful and medical treatment of the underlying Crohn's disease proved subsequently successful, with clinical and biological parameters of inflammation returning to normal within 10 days. Seven months after surgery, the patient reports one episode of diarrhoea per week; her blood tests are normal and azathioprine was reduced to 100 mg OD.

## Discussion

We report the case of a young woman who presented with extension of ileal Crohn's disease lesions into adjacent Meckel's diverticulum, resulting in perforation and abscess formation. A similar case was previously described in an elderly patient who did not have active Crohn's disease of the ileum, either distal or proximal to the diverticulum [7]. In our patient, there are three lines of evidence suggesting that perforated Meckel's diverticulitis is directly related to Crohn's disease: 1) active Crohn's disease was present proximal and distal to the diverticulum; 2) presence of transmural inflammation and giant cell granulomas in the surgical specimen; and 3) absence of heterotopic gastric mucosa within the resected diverticulum.

Two surgical strategies were conceivable in this peculiar situation: either an ileocaecectomy or a Meckel's diverticulectomy. The first option offered the opportunity to perform a relatively safe ileo-caecal anastomosis, but required an extensive (80 cm) small bowel resection in a young patient. We chose to preserve as much as possible of her small bowel and limited the resection to the Meckel's diverticulum. This alternative, in a septic environment, on top of active Crohn's disease, carried the risk of staple line disruption, and would not have been our first choice in an older patient. Two *sine qua non *conditions for performing a Meckel's diverticulectomy were met in this specific case: 1) the absence of stricturing disease distal to the perforation; and 2) the fact that the diverticulum was quite long with a relatively healthy base, which appeared suitable for linear stapling. Obviously, great care was taken to fire the stapler alongside the axis of the small bowel in order to avoid any reduction of the bowel endoluminal diameter.

## Conclusion

This case illustrates how Crohn's disease may extend into adjacent Meckel's diverticulum and cause perforated diverticulitis. In this young patient, however, this unusual combination was a blessing in disguise, the septic complication being taken care of without any small bowel resection.

## Abbreviations

BD: twice daily; CT: computed tomography; OD: once daily; TID: three times daily

## Competing interests

The authors declare that they have no competing interests.

## Authors' contributions

FS and PG analyzed and interpreted the data, operated on the patient and wrote the manuscript. PS was involved in the endoscopic and gastrointestinal follow-up of the patient before and after surgery. TMK performed the histological examination. PM was a major contributor in writing the manuscript. All authors read and approved the final manuscript.

## Consent

Written informed consent was obtained from the patient for publication of this case report and accompanying images. A copy of the written consent is available for review by the Editor-in-Chief of this Journal.
